# Health-related quality of life, motivational regulation and Basic Psychological Need Satisfaction in Education Outside the Classroom: an explorative longitudinal pilot study

**DOI:** 10.1186/s12889-021-12450-9

**Published:** 2022-01-08

**Authors:** Jan Ellinger, Filip Mess, Simon Blaschke, Christoph Mall

**Affiliations:** Department of Sport and Health Sciences, Associate Professorship of Didactics in Sport and Health, Technical University of Munich, Georg-Brauchle-Ring 60/62, 80992 Munich, Germany

**Keywords:** Children, Education outside the Classroom, Basic Psychological Needs, Well-being, Health-related quality of life, School Motivation, Linear mixed-effect models

## Abstract

**Background:**

Given a suboptimal state of mental health among children, an urgent need exists to seek approaches related to health promotion in this population’s settings, such as in schools. Increased health-related quality of life (HRQoL) and improved school motivation could be crucial for children’s mental health. Based on *self-determination theory*, paths can be identified that could lead to such improvements by strengthening the basic psychological needs (BPN). This study aimed to examine the impact on and the relationships among HRQoL, school motivation and BPN within the promising concept of *education outside the classroom* (EOtC).

**Methods:**

In this exploratory study, we employed a between-subjects cohort study design with no blinding or randomisation. We surveyed fifth graders (mean = 10.1 years) attending EOtC (experimental group [EG], n = 25) and normal indoor lessons (control group, [CG], n = 41) at the beginning (T1) and end (T2) of a semester. We used the translations of validated questionnaires and established linear mixed-effects models to evaluate whether the students in EOtC show higher scores of HRQoL and school motivation and, whether the satisfaction of BPN of autonomy (PAut), competence (PCom), social relatedness with classmates (PSRC) and teachers (PSRT) show associations with these outcomes.

**Results:**

Regarding intrinsic and identified motivational regulation, results showed significant increases over time in the overall sample and significant higher scores in the EG than in the CG. For HRQoL, no group differences were found, but a significant decrease over time in the EG. Regarding possible associations between the outcomes and BPN, such could only be found between HRQoL and PSRC, but not for the other BPN and not for motivational regulation and BPN.

**Conclusions:**

Without having been able to explain this on the basis of increased BPN values, our results show that EOtC can support improvements in specific regulation types of school motivation. This could contribute to an improvement in the mental health situation in children, as school represents a major stressor for them. Future steps in terms of researching HRQoL in this setting are discussed, as this pilot study does preliminary work for necessary examinations, e.g. in structural equation approaches.

**Supplementary Information:**

The online version contains supplementary material available at 10.1186/s12889-021-12450-9.

## Background

Today, large proportions of the child and adolescent population exhibit mental health issues. In a recent study, 12.8% of children (aged 6–11 years) showed mental health problems [1]. Looking at the population of children, adolescents and young adults under 25 years of age, 14.3% are officially diagnosed with depression [2]. At the beginning of adolescence, individuals are particularly susceptible to effects on their mental health and well-being because a large number of psychological and physical changes and developments take place during this time [3]. Anniko et al. [4] identified school pressure as the source of mental health problems over all other considered sources (e.g. arguments at home or romantic relationships) in a longitudinal study. Therefore, a suitable entry point for interventions tackling these issues seems to be the school because children and adolescents spend a large part of their waking hours there [5, 6], and there is the tendency to even increase the time per day spent at school, at least in European countries [7, 8]. Subsequently, current studies should, on the one hand, verify the effectiveness of developed interventions on mental health in the school setting (e.g. [9, 10]); however, on the other hand, also explain the effects in detail by using specific theories and models, such as the *health-action-process-approach* in the field of health behaviour change [11] or *achievement goal theory* in the research of intrinsic motivation [12]. Additionally, *self-determination-theory* (SDT) seems suitable for the considerations of health-related issues in general, which is the reason we will focus on this theory in the following chapter. A meta-analysis led to the assumption that a foundation of SDT in particular could possibly promise success regarding physical and mental health because the results suggest a positive association between SDT-based constructs and indicators of mental health [13], such as well-being and health-related quality of life (HRQoL) [14, 15]. According to our understanding, well-being and HRQoL have significant overlaps from a psychological perspective and are not necessarily distinguishable from each other [16, 17]. In this article, we always use the terminology of the original authors when reporting on previous studies. With the increased use of HRQoL in publications and surveys in recent years [18], we use this term when describing our own considerations.

### Background on Self-determination Theory

Within SDT, as a first central element, authors Ryan and Deci [19, 20] distinguish different types of motivational regulation: intrinsic, integrated, identified, introjected and external motivation. In addition, explicit amotivation can be seen as a special form in close connection with these types, which expresses the lack of any motivation with regard to a target behaviour. An intrinsic type of motivation here corresponds to maximally self-determined motivation, which is the reason these two terms have a great degree of overlap but must be distinguished at the theoretical level [20]. External motivation as the second extreme of the continuum, on the other hand, is not based on personal value, but rather on external rewards or punishments that are associated with the corresponding behaviour. The types of integrated, identified and introjected motivational regulation each set their own semantic priorities, but all fall between these two extremes [20]. In order to further summarize these types of motivational regulation, they can be hierarchically described in the sense of Ryan and Deci with intrinsic, integrated and identified motivation as self-determined types and and introjected and external motivation as controlled motivational regulation types, besides amotivation [19].

As the second central element of SDT, basic psychological needs (BPN) are described as needs that are equal to all persons across gender, culture and time [21]. Three BPN dimensions are distinguished: autonomy, competence and social relatedness, which, in the specific context of school, can be subsequently divided into social relatedness with teachers on the one hand and classmates on the other [19]. In the context of the practical research application of these dimensions, the subscales are often marked as ‘perceived’ (perceived autonomy = PAut; perceived competence = PCom; perceived social relatedness with teachers = PSRT; perceived social relatedness with classmates = PSRC). Regarding BPN, the needs for PAut and PCom are most closely linked [22]. SDT is considered an established theory of motivation in various areas, such as research in work organisations [23] or environmental education [24].

The majority of SDT-informed health-domain interventions focus on BPN in relation to other variables, such as mental health outcomes [25], and it seems quite possible to cause a (positive) change in SDT elements (e.g. level of satisfaction of BPN) through interventions [13]. The reported dimensions of the BPN can also, in essence, be directly applied to the school setting [26, 27].

### State of Research on Self-determined Motivation, HRQoL and BPN in schools

SDT holds possible starting points for improving HRQoL and motivational regulation in students. Both could be at least partly explained by the satisfaction of BPN. Those relationships between the degree of satisfaction of specific BPN and these practical relevant outcomes of HRQoL and motivational regulation can be well argued, as described in the following.

#### Relationship Between HRQoL and BPN

If the satisfaction of the BPN is not sufficient, comprehensive health is difficult to imagine [21], which is why it can be argued that an increase in the satisfaction of BPN can already lead to improvements in HRQoL [28, 29]. Although some studies suggest the great importance of social relatedness in particular for HRQoL it can be assumed that all three BPNs can have a positive effects here as there is no clear trend towards a superior importance of one of the BPNs over the others (e.g. [30–32]). In the past, Tian et al. identified a relationship between BPN and school-related well-being with the BPN as a mediator between school-related social support and well-being [33, 34]. In the context of specific school subjects, the possible impact of BPN-satisfaction on HRQoL was pointed out [35, 36]. A significant decrease in HRQoL is observed with an increase in the age of the students [37].

#### Relationship Between Motivational Regulation and BPN

Empirical evidence suggests that satisfaction of the BPN correlates with internalisation and intrinsic motivation in adults [38]. In particular, the needs of autonomy and competence could be considered as tenets and predictors of a more self-determined type of motivational regulation [39]. Of course, these relationships are also of particular interest in institutions that are characterised by learning and teaching, such as in universities and schools, as the BPN have already been identified as an essential factor in motivation [40–42]. Studies that carried out a detailed breakdown of both the BPN and different motivational regulation types were able to show on the basis of larger samples within the German and Australian school setting that in particular autonomy and competence show an positive influence on the intrinsic and identified motivational regulation [43, 44]. In analogy to the observations on HRQoL, a steady decline of motivation can be observed during adolescence, which is predicted by a decline in the satisfaction of the BPN [45].

The findings show that satisfaction of the BPN could be crucial to both motivational regulation and the HRQoL of students. For HRQoL all BPN seem of importance, while for motivation PCom and particularly PAut seem to be crucial factors [21, 39]. Beyond the association of BPN and HRQoL, an increase in self-determined and intrinsic motivation could also have a positive impact on HRQoL on the long run [46], as intrinsic motivation plays a unique role in predicting academic performance [47]. This in turn can be related to subjective well-being based on the results of a large-scale meta analysis [48]. Certain school concepts could start at this point and enable improvements in the corresponding constructs of motivational regulation and HRQoL aside the satisfaction of BPN.

### Education Outside the Classroom and the Impact on HRQoL

The concept of *education outside the classroom* (EOtC) could be of great value for improving HRQoL via the described mechanisms in students. EOtC can be defined as regular teaching that takes place in natural, industrial or cultural locations in accordance with the curriculum. Which methods are used and with which aids or agreements the learning contents are conveyed are the responsibility of the school or the teacher [49, 50]. In Scandinavia, the concept is applied in approximately 20% of all public and private schools [51], whereas in Germany, similar to many other countries, only isolated attempts by individual schools have been observed to date; however, there is a lot of rising interest toward this concept [52]. Because EOtC appears promising from the perspectives of health and learning, it has already moved into the extended focus of research. In their review, Becker and colleagues summarised and critically appraised first findings regarding the effects of EOtC on social, learning and health dimensions [53]. Recent publications indicate a connection between EOtC and increased physical activity [54–56], a gain in peer affiliations [57] and inconsistent results on the influence on academic performance in school subjects [58, 59].

The evidence on concrete effects of EOtC on HRQoL is still rather incomplete. So far, initial findings provide evidence that the psychosocial well-being of children in EOtC could particularly be enhanced by an increase in prosocial behaviour [60]. Links to possible effects on HRQoL also arise from findings regarding stress. Thus findings show that the profile of cortisol secretion in students taught outdoors may be closer to what is considered a (stress-related) healthy pattern and thus could contribute to an improvement in the stress-related mental health situations in the student population [61]. Breaks and rest periods in particular could also have a positive effect in this respect [62]. Effects on the learning process and school motivation are also still rare in EOtC, but there are also first tendencies. For example, the descriptions of students taught outdoors showed that the learning process was not even perceived as learning in the formal sense by the students [63]. Methodologically well-structured studies also show the importance of the EOtC as a possible buffer against a drop in the students’ intrinsic motivation over the course of a school year [64].

As previously pointed out, such effects could be at least partly due to the satisfaction of the BPN. Following that, the question arises as to whether EOtC can influence levels of satisfaction of BPN in students. Regarding PAut, the possibility of choices within an environment of structure is considered particularly beneficial [65, 66]. EOtC offers a wide range of potential choices capable of influencing PAut (cf. Supplementary Material), such as when choosing a partner for tasks, the place where the students want to learn, which writing underground they use (e.g. forest soil, tree trunk, clipboard) or in which order they work themselves through working stations. The balance of a controlled structure plays a decisive role in patterns of autonomy-supportive teaching [67], which is a particular challenge but also an opportunity for the teachers, particularly in initially unstructured natural environments [68]. Therefore, it seems quite reasonable to assume that autonomy is particularly promoted by EOtC. Additionally, a promotion of the PCom seems promising through EOtC. The introduction of EOtC is disruptive in the extreme, which is the reason why all elements of instruction are bound to be questioned. Because teachers in EOtC rely heavily on the engagement, co-operation and discipline of their students, it is quite conceivable that competence-supporting strategies, such as incorporating students’ opinions, will be increasingly applied in EOtC. Additionally, a learning environment in nature offers competence-supportive characteristics to a special degree [69], such as the necessity of pointing out concrete plans of learning locations and contents because in EOtC, unlike in classical indoor settings, these are not to be considered as set. In addition, the will to acquire competences could be increased among the students because of the high relevance to everyday life [70]. When considering the BPN of social relatedness, the EOtC also reveals a wide range of possibilities for positive effects. Already the intensive engagement with and in nature in couple constellations or groups seems to lead to positive effects in social interaction [71, 72]. Compared with a classroom setting, the EOtC offers very obvious opportunities for social interaction at least in natural environments. Because spatial limitations are almost completely eliminated, one can speak of a new form of movement in space, from which new social interactions can also arise [73], which could in turn be reflected in increased social relatedness.

Taken together, there are indications suggesting that EOtC may improve HRQoL, in parallel with or conditioned by improvements in self-determined motivational regulation and satisfaction of the BPN. However, the corresponding findings come from different studies and cannot be directly linked to each other or were not gained from studies with regularly conducted EOtC. Therefore our pilot study aims to gain a deeper understanding of individual processes of these in EOtC. For this purpose we set up the following hypotheses to explore this in an step-by-step manner: (a) EOtC-class and control group differ in terms of their scores of HRQoL over time; (b) EOtC-class and control group differ in terms of their scores of motivational regulation types over time; (c) EOtC-class and control group differ in terms of the association of HRQoL and BPN; (d) EOtC-class and control group differ in terms of the association of motivational regulation types and BPN.

## Methods

### Intervention

Our study began in the summer of 2019 in co-operation with a secondary school located in southwest Germany. The co-operating school incorporated EOtC in the school year 2019–2020 into their curriculum for the first time. The underlying teaching concept was based on practical knowledge of EOtC gained by teachers from Germany and was elaborated before the beginning of the school year. The EOtC-class was curriculum compliant and was implemented every Thursday from 08:30 a.m. to 12:55 p.m. in a nearby forest. After an initial lesson in the school facilities, which was used for tasks, such as to check attendance and prepare important contents, students and teachers walked to the forest (estimated 15 min of walking). Onsite, various locations were used for teaching. During the following lessons, two teachers held two different outdoor classes selecting from the school subjects of German and Biology. At the end of each school day, teachers and students made their way back to the school together. Additional details about the teaching situation in the EOtC in general and within our study are presented in the supplementary material.

### Study Design

In addition to the EOtC-receiving experimental group (EG), the secondary school contained three other fifth-grade classes, which received regular indoor teaching in the corresponding subjects and served as the control group (CG). In this pilot study, we used a between-subjects, prospective cohort study design with no blinding or randomisation. Initially, we planned four measurement points spread over the entire school year. With the outbreak of the COVID-19 pandemic and the resulting school closures throughout Germany, this plan could not be fully implemented. Therefore, only two measurement points (T1, T2) were conducted at the start (October) and end (February) of the first semester. This represents four weeks of EOtC until T1 and 17 weeks until T2 for the EG.

### Participants

 We contacted a total of 100 students and their parents from all four fifth-grade classes of the school year 2019–2020 in the run-up of our study. Based on the declarations of consent of the students and parents, we were able to enrol 70 students who attended the school at that time in the fifth grade. In the EG, all students agreed to participate in our study. During the two enquiries, two students each had to be removed from the EG and the CG because of a change of school or withdrawal of consent. Our analysis therefore contained 66 students. Our EG comprised 25 students (female, n = 11; male, n = 14), and the CG comprised of 41 students (female, n = 15; male, n = 26). With illness or other reasons of absence, we were not able to collect data from every student at every time point. The participants had a mean age of 10.1 years at T1, with the minimum and maximum ages of 9 and 11 years. Enrolment data and descriptive statistics are presented in the chapter ‘Results’ and the Supplementary Material.

### Data Collection

Using a combined questionnaire, the measures necessary to test the hypotheses and demographic information were collected. The *Basic Psychological Needs Scale* (*BPNS)* was used for the purpose of assessing BPN. The *BPNS* was originally developed by La Guardia, Ryan, Couchman and Deci [74], and for application in our study, we used the German translation by Hanfstingl et al. [75]. The *BPNS* comprises 24 items on a 5-point Likert scale and contains the subscales of PAut, PCom, PSRT and PSRC. Higher scores indicate better satisfaction of BPN. For our data, all subscales showed acceptable to good values for Cronbach’s alpha (PAut: α = 0.82; PCom: α = 0.80; PSRT: α = 0.75; PSRC: α = 0.62).

The *KIDSCREEN-27* Questionnaire [76] was of choice to determine the HRQoL and is suitable for assessing the subjective health and well-being of children aged 8–18 years [77]. The *KIDSCREEN-27* is a validated short form of the *KIDSCREEN-52* and has already been used in numerous studies in similar age groups [78, 79]. The sum score of the items (α = 0.83) was transformed to Rasch person parameters by using an algorithm according to the manual [76]. This results in a mean score of 50 with SD = 10 for the children in the reference group [80]. Higher total scores indicate a better HRQoL.

The *Academic Self-Regulation-*Questionnaire *(SRQ-A)* was used to assess motivational regulation in school [81]. The *SRQ-A* is a 32-item questionnaire (4-point Likert scale) that measures students’ attitudes towards schoolwork. Particularly in the field of EOtC research, the *SRQ-A* seems to be a common and reliable tool as shown by its use in the studies of Bølling et al. [64] and Dettweiler et al. [69]. In our study, the German validated version of the *SRQ-A* by Kröner et al. [82] was used. The SRQ-A provides results for the individual subscales of intrinsic (α = 0.80), identified (α = 0.82), introjected (α = 0.75) and external motivational regulation (α = 0.75), whereas higher scores indicate a higher degree in the corresponding motivational regulation type.

In addition to the aforementioned measures, within this project, other parameters were collected from the students. These parameters will be addressed in publications with a different thematic focus. More details on the entire project design can be found on the Center for Open Science: https://osf.io/f32 kg/.

### Statistical Analysis

For our main analysis, we used a linear mixed-effects model (LMM) design to account for the characteristics of our data [83]. In order to obtain information not only on whether the BPN are positively or negatively associated with the outcomes, but also on whether the satisfaction of the BPN in the EG differs significantly from that in the CG, we additionally conducted a multivariate analysis of variance (MANOVA) for this purpose. In doing so, we defined the four facets of the BPN as dependent variables and the groups as independent variables. For the LMMs, based on the literature, BPN (resp. the relevant subscales) were defined as fixed factors. The explained variance over time of the inherent individual determinants was of particular interest. Consequently, we defined the individual as a random factor. The metrics of the dependent variables were left on their T-scores in the case of HRQoL, as recommended by the authors [76]. In the case of motivational regulation, we used the raw average values of the motivational regulation types. We screened for multivariate outliers by using Mahalanobis distance [84] but found none. Subsequently, we decided to keep the few univariate outliers in our data because we have no evidence that the values were skewed by measurement errors or the inclusion of someone not in the target group. Because our outcome scores do not have meaningful zero values in their initial form, centring was performed for them in advance of the analysis. We report estimates (regression coefficients), their confidence intervals (CI 95%) and Cohen’s d (*d)* as indicators for the effect sizes as wells as standard deviations. We also consulted R-squared (*R*^*2*^) [85] to determine the proportion of variability explained by fixed and random factors in the different models. The initial general model of our analysis can be represented as follows for the enquiry $i$ of person $j$ of gender $k$ in group $l$:

$${y}_{ijkl}= {\beta }_{0}+{(\beta }_{1}+{r}_{1k})*{x}_{ijkl}+ {\beta }_{i}+{\beta }_{ij}+ {\beta }_{ijk}+ {\beta i}_{jkl}+ {\epsilon }_{ijk}$$

${\beta }_{0}$ is the intercept, and ${\beta }_{1}$ represents the slopes. $x$ represents the fixed factors of BPN. The residuals$r$ and $\epsilon$ are also zero mean normally distributed random variables with covariance matrix dependent on the situation as described on the corresponding levels. This approach allowed us to determine the general influence of the satisfaction of the BPN while considering groups, gender and time point of enquiry and random effects because of the repeated measurement of the same individuals. The model fit was evaluated using the AIC criterion and restricted maximum likelihood ratio tests. Based on the presented previous findings, we constructed five models to account for our hypotheses. Four models recorded the motivational regulation types as a dependent variable based on the BPN (Intrinsic: Model 1; Identified: Model 2, Introjected: Model 3; External: Model 4). In the last model (Model 5), HRQoL was implemented as a dependent variable, and the BPN were again implemented as independent variables. For all models the corresponding variant of the model without interaction effects of Group and Gender alongside all other variables showed better model fits (cf. Supplementary Table 1). Heteroskedasticity was rejected by visual inspection for all of the five models and confirmed by Levene´s test [86]. Multicollinearity could be rejected by calculating the variance inflation factor for our respective independent variables and their subscales and following the recommendations by Wooldridge [87]. This was particularly necessary in view of possible content overlaps in KIDSCREENs subscale “Social Support and Peers” with PSRC. The software *RStudio* (Version 1.3, RStudio Inc., Boston, USA) was used for data analysis [88]. The imputation of missing values was performed by predictive mean matching based on predicted values, which also take care of the random effects, by using the method ‘2l.lmer’ of the package ‘MICE’ [89–91]. In the analysis of the five mixed models, we used the software package ‘nlme’ [92], as well as for our MANOVA.

## Results

**Fig. 1:**
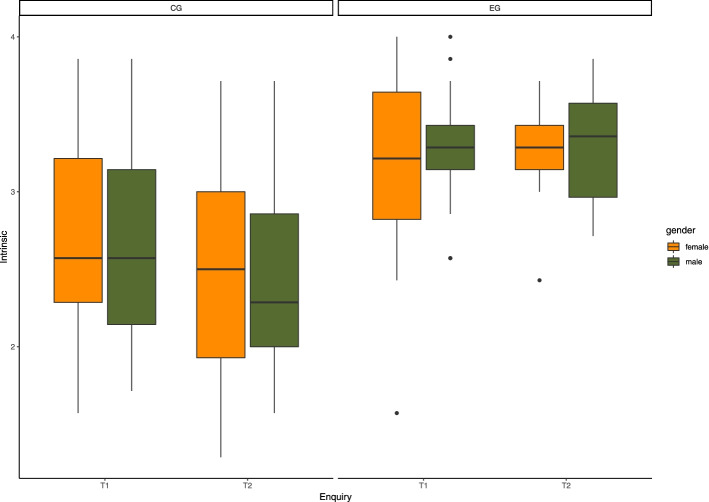
Intrinsic motivational regulation between groups (EG, CG), gender (female, male) and enquiries (T1, T2)

Data reflecting the first time point at the beginning of the school year could be used from 66 participants (EG: 25; CG: 41) and at the second time point from 64 participants (EG: 23; CG: 41). In total, the data of 25 girls and 41 boys were included. The discrepancy in the EG is due to absence from school because of illness or other reasons. Table 1 provides an overview of the average values, standard deviations and confidence intervals achieved for the outcomes of HRQoL and the four motivational regulation types collected at the corresponding enquiry in the individual groups. The descriptive data already indicate that the EG could have advantages in intrinsic and identified motivational regulation (cf. Fig. 1 and Supplementary Fig. 2). For HRQoL, EG shows higher scores at T1. For the facets of BPN, at first glance, we see comparable values between the groups and no apparent trends over time (cf. Supplementary Table 3).

**Table 1:** Statistical characteristics for the outcomes of HRQoL and motivational regulation types between groups, genders and enquiries

			T 1					T 2				
			HRQoL	Intr.	Ident.	Intro.	Ext.	HRQoL	Intr.	Ident.	Intro.	Ext.
**EG**	**Girls**	**M**	54.18	3.00	3.33	2.58	2.57	56.53	3.39	3.45	2.91	2.61
		**SD**	9.84	0.56	0.31	0.70	0.69	7.79	0.22	0.44	0.63	0.62
		**CI**^**a**^	48.36 / 60.0	2.44 / 3.56	3.02 /3.64	1.88 / 3.28	1.89 / 3.26	51.92 / 61.14	3.17 / 3.61	3.01 / 3.90	2.28 / 3.53	1.99 / 3.24
	**Boys**	**M**	59.51	3.44	3.54	3.26	2.88	57.14	3.39	3.27	2.79	2.57
		**SD**	7.70	0.41	0.38	0.50	0.48	8.35	0.34	0.52	0.67	0.53
		**CI**^**a**^	55.48 / 63.54	3.03 / 3.85	3.15 / 3.92	2.76 /3.76	2.39 / 3.36	52.76 / 61.52	2.85 / 3.53	2.76 / 3.79	2.12 / 3.46	2.04 / 3.11
	**All**	**M**	59.58	3.23	3.44	2.93	2.73	56.85	3.29	3.46	2.85	2.59
		**SD**	9.31	0.53	0.37	0.69	0.61	8.10	0.30	0.49	0.65	0.58
		**CI**^**a**^	52.93 / 60.23	2.69 / 3.76	3.07 / 3.81	2.24 / 3.62	2.12 / 3.34	53.68 / 60.02	2.98 / 3.59	2.87 / 3.85	2.19 / 3.50	2.01 / 3.17
**CG**	**Girls**	**M**	57.03	2.84	3.16	2.93	2.83	54.25	2.37	2.91	2.73	2.83
		**SD**	8.48	0.69	0.64	0.60	0.49	9.23	0.56	0.56	0.76	0.71
		**CI**^**a**^	52.74 / 61.32	2.15 / 3.53	2.52 / 3.81	2.33 / 3.53	2.34 / 3.33	49.58 / 58.92	1.82 / 2.94	2.35 / 3.47	1.97 / 3.48	2.11 / 3.54
	**Boys**	**M**	55.65	2.52	3.01	2.79	2.88	55.44	2.54	2.97	2.91	2.83
		**SD**	5.32	0.59	0.67	0.51	0.48	8.78	0.58	0.57	0.62	0.58
		**CI**^**a**^	53.61 / 57.69	1.94 / 3.11	2.34 / 3.67	2.27 / 3.30	2.40 / 3.35	52.06 / 58.82	1.96 / 3.12	2.39 / 3.54	2.29 /3.53	3.25 / 3.41
	**All**	**M**	56.15	2.64	3.06	2.84	2.86	55.01	2.58	2.95	2.84	2.83
		**SD**	6.69	0.64	0.66	0.55	0.49	8.96	0.58	0.57	0.68	0.63
		**CI**^**a**^	54.1 / 58.2	1.99 / 3.28	2.40 / 3.72	2.29 / 3.39	2.37 / 3.35	52.27 / 57.75	1.90 / 3.05	2.37 / 3.52	2.16 / 3.52	2.20 / 3.47
**All**		**M**	56.32	2.88	3.24	2.86	2.78	55.67	2.82	3.15	2.89	2.79
		**SD**	7.79	0.67	0.56	0.61	0.52	8.71	0.63	0.58	0.67	0.62
		**CI**^**a**^	54.44 / 58.2	2.21 / 3.55	2.68 / 3.80	2.25 / 3.47	2.26 / 3.30	53.57 / 57.77	2.19 / 3.45	2.57 / 3.73	2.22 / 3.56	2.16 / 3.41

*HRQoL* Health-related quality of life; *Intr.* Intrinsic motivational regulation (MR); *Ident* Identified MR; *Intro.* Introjected MR; *Ext.* External MR; *M* Mean*; SD* Standard deviation*; CI* Confidence interval*;* ^a^ *95%, Lower / Upper CI*

The first inferential result of our MANOVA is that there are group differences in the level of satisfaction of the BPN (cf. Supplementary Table 2). Thus, the EG shows an advantage in PAut (p < 0.05) and PCom (p < 0.01), while the CG reveals significantly higher values in PSRC (p < 0.001). These results will later serve as a basis for the interpretation of the main analysis.

Our LMM analysis revealed the following results. An overview of the results of all of our models is given in Table 2. As per our first hypothesis, we expected group differences in HRQoL over time, possibly in favour of EG. However, our analysis of the corresponding model (${r}^{2}=0.61$) could not find any group differences (p = 0.215, ${{\upeta }}^{2}$ = 0.31, β = 2.49). What was noticeable, however, was a significant decrease in the HRQoL values of the EG at T2 (p = 0.016, ${{\upeta }}^{2}$ = -0.71, β = -5.57). With regard to our second hypothesis, some significant results were obtained: The EG shows significantly higher values than the CG in intrinsic (p = 0.003, ${{\upeta }}^{2}$ = 0.79, β = 0.51) and identified (p = 0.040, ${{\upeta }}^{2}$ = 0.54, β = 0.33) motivational regulation, while no differences were evident in the external (p = 0.220, ${{\upeta }}^{2}$ = -0.31, β = -0.22) and introjected (p = 0.957, ${{\upeta }}^{2}$ = 0.01, β = 0.01) types.

**Table 2:** Results of the analysis of the five different models regarding p-values (p), effect sizes (η^2^), regression coefficients (β) and confidence intervals (CI)

	p	η^**2**^	β	CI^**a**^	p	η^**2**^	β	CI^**a**^
	**Model 1**^**b**^				**Model 2**^**b**^			
**PAut**	0.357	0.27	0.17	-0.19 / 0.53	0.229	0.35	0.21	-0.14 / 0.56
**PCom**	0.155	0.42	0.26	-0.10 / 0.61	0.940	0.02	0.01	-0.33 / 0.35
**PSRC**	0.449	0.22	0.11	-0.18 / 0.40	0.730	0.10	0.05	-0.23 / 0.33
**PSRT**	0.559	-0.17	-0.08	-0.37 / 0.20	0.710	0.11	0.05	-0.22 / 0.33
**Male**	0.887	-0.04	-0.02	-0.33 / 0.28	0.800	0.03	0.02	-0.27 / 0.31
**EG**	**0.003**	0.79	0.51	0.18 / 0.83	**0.040**	0.54	0.33	0.02 / 0.64
**T2**	**0.007**	0.81	2.49	0.71 / 4.28	**0.034**	0.63	1.84	0.14 / 3.54
**PAut:EG**	0.548	0.25	0.46	-1.09/ 2.00	0.738	0.14	0.23	-1.22 / 1.70
**PCom:EG**	0.419	0.34	0.59	-0.89 / 2.08	0.706	0.16	0.26	-1.14 / 1.67
**PSRC:EG**	0.285	-0.46	-0.71	-2.04 / 0.63	0.519	0.27	0.40	-0.86 / 1.66
**PSRT:EG**	0.560	0.25	0.47	-1.18 / 2.13	0.819	-0.09	-0.18	-1.74 / 1.39
**Male:EG**	0.476	0.28	2.71	4.86 / 10.28	0.558	0.15	2.11	-5.04 / 9.26
**T2:EG**	0.116	0.46	0.34	-0.09 / 0.76	0.301	0.30	0.21	-0.19 / 0.61
**PAut:T2**	0.505	-0.19	-0.18	-0.70 / 0.35	0.460	-0.26	-0.23	-0.74 / 0.27
**PCom: T2**	0.161	-0.41	-0.39	-0.94 / 0.16	0.459	-0.22	-0.20	-0.73 / 0.33
**PSRC:T2**	0.421	-0.23	-0.14	-0.49 / 0.21	0.807	-0.07	-0.04	-0.38 / 0.29
**PSRT:T2**	0.799	0.07	0.05	-0.34 / 0.44	0.785	-0.08	-0.05	-0.43 / 0.32
**Male:T2**	0.116	-0.10	-0.07	-0.47 / 0.33	0.645	0.13	0.09	-0.29 / 0.47
	**Model 3**^**b**^				**Model 4**^**b**^			
**PAut**	0.390	0.25	0.18	-0.24 / 0.60	0.731	0.09	0.06	-0.24 / 0.60
**PCom**	0.304	0.30	0.21	-0.20 / 0.62	0.297	0.30	0.18	-0.20 / 0.60
**PSRC**	0.463	-0.21	-0.12	-0.45 / 0.21	0.487	-0.20	-0.10	-0.46 / 0.21
**PSRT**	0.771	-0.08	-0.05	-0.38 / 0.28	0.794	-0.07	-0.03	-0.38 / 0.28
**Male**	0.126	0.39	0.27	-0.07 / 0.61	0.131	0.39	0.23	-0.08 / 0.61
**EG**	0.957	0.01	0.01	-0.35 / 0.37	0.220	-0.31	-0.20	-0.35 / 0.37
**T2**	0.185	0.39	1.42	-0.70 / 3.55	0.963	-0.01	-0.04	-0.70 / 3.55
**PAut:EG**	0.105	0.70	1.31	-0.29 / 2.89	0.448	0.32	0.52	-0.87 / 1.91
**PCom:EG**	0.977	-0.01	-0.02	-1.55 / 1.51	0.716	0.15	0.24	-1.11 / 1.59
**PSRC:EG**	0.985	-0.01	-0.01	-1.39 / 1.36	0.250	0.49	0.69	-0.52 / 1.87
**PSRT:EG**	0.656	-0.19	-0.37	-2.08 / 1.34	0.154	-0.61	-1.06	-2.56 / 0.43
**Male:EG**	0.111	0.41	6.33	-1.49 / 14.14	0.717	0.09	1.24	-5.59 / 8.07
**T2:EG**	0.970	-0.01	-0.01	-0.52 / 0.01	0.799	0.07	-0.06	-0.52 / 0.50
**PAut:T2**	0.714	-0.10	-0.11	-0.73 / 0.50	0.240	0.34	0.32	-0.73 / 0.50
**PCom: T2**	0.318	-0.29	-0.32	-0.97 / 0.32	0.067	-0.54	-0.53	-0.97 / 0.32
**PSRC:T2**	0.706	0.11	0.08	-0.34 / 0.50	0.202	0.37	0.24	-0.34 / 0.50
**PSRT:T2**	0.762	0.09	0.07	-0.39 / 0.53	0.840	0.06	0.04	-0.39 / 0.53
**Male:T2**	**0.042**	-0.60	-0.50	-0.98 / -0.02	**0.031**	-0.07	-0.47	-0.98 / -0.01
	**Model 5**^**b**^							
**PAut**	0.300	0.64	4.77	0.46 / 9.08				
**PCom**	0.175	0.39	2.85	-1.31 / 7.02				
**PSRC**	**0.007**	0.79	4.78	1.35 / 8.22				
**PSRT**	0.074	-0.52	-3.07	-6.44 / 0.31				
**Male**	0.321	-0.25	-1.88	-5.65 / 1.88				
**EG**	0.215	0.31	2.49	-1.48 / 6.46				
**T2**	0.749	0.09	3.14	-16.46 / 22.74				
**PAut:EG**	0.291	-0.44	-8.02	-23.35 / 7.31				
**PCom:EG**	0.449	0.31	5.49	-9.24 / 20.21				
**PSRC:EG**	0.994	-0.01	-0.05	-13.43 / 13.34				
**PSRT:EG**	0.901	0.05	1.02	-15.70 / 17.76				
**Male:EG**	0.578	0.14	21.47	-55.27 / 98.22				
**T2:EG**	**0.016**	-0.71	-5.57	-10.38 / -1.12				
**PAut:T2**	0.314	-0.29	-2.92	-8.69 / 2.85				
**PCom: T2**	0.904	-0.03	-0.37	-6.52 / 5.77				
**PSRC:T2**	0.385	-0.25	-1.69	-5.58 / 2.19				
**PSRT:T2**	0.062	0.54	4.18	-0.22 / 8.59				
**Male:T2**	0.108	0.47	3.53	-0.80 / 7.86				

^a^95%, Lower / Upper confidence interval; ^b^Dependent variable (DV) in Model 1: Intrinsic motivational regulation; DV Model 2: Identified motivational regulation; DV Model 3: Introjected motivational regulation; DV Model 4: External motivational regulation; DV Model 5: HRQo

**Fig. 2:**
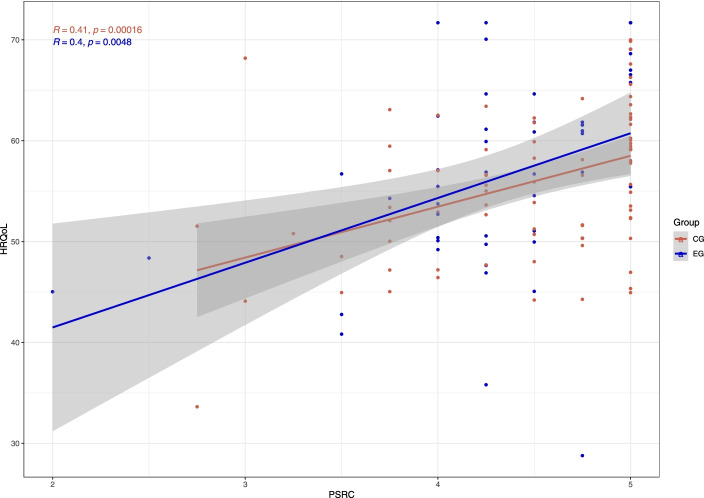
Relationship of PSRC and HRQoL between groups (EG, CG) over all genders (female, male) and enquiries (T1, T2)

Across both groups, there was also a significantly positive development over time for the intrinsic (p = 0.007, $${{\upeta }}^{2}$$ = 0.81, β = 2.49) and the identified (p = 0.034, $${{\upeta }}^{2}$$ = 0.63, β = 1.84) type. Especially boys showed a significant drop over time in the external (p = 0.031, $${{\upeta }}^{2}$$ = -0.07, β = -0.47) and introjected (p = 0.042, $${{\upeta }}^{2}$$ = -0.60, β = -0.50) types. Measured with the values of R-squared, the models which included intrinsic ($${r}^{2}=$$$$0.46$$) and identified motivational regulation ($${r}^{2}=$$$$0.37$$) as outcomes showed the comparatively best fit within motivation (Introjected: $${r}^{2}=$$$$0.19$$; External: $${r}^{2}=$$$$0.21$$). With regard to our third hypothesis, a significantly positive relationship between HRQoL and PSRC (p = 0.007, $${{\upeta }}^{2}$$ = 0.79, β = 4.78) could be stated (cf. Fig. 2), but not for the other BPN (PAut: p = 0.300, $${{\upeta }}^{2}$$ = 0.64, β = 4.78; PCom: p = 0.175, $${{\upeta }}^{2}$$ = 0.39, β = 2.85; PSRT: p = 0.074, $${{\upeta }}^{2}$$ = -0.52, β = -3.07) or in interaction with affiliation to EG (PAut: p = 0.291, $${{\upeta }}^{2}$$ = -0.44, β = -8.02; PCom: p = 0.449, $${{\upeta }}^{2}$$ = 0.31, β = 5.49; PSRC: p = 0.994, $${{\upeta }}^{2}$$ = -0.01, β = -0.05; PSRT: p = 0.901, $${{\upeta }}^{2}$$ = 0.05, β = 1.02).

With regard to the motivational regulation in the context of our last hypotheses, no significant relationships could be determined, neither for the individual BPN itself (e.g. in relation with intrinsic motivational regulation: PAut: p = 0.357, ${{\upeta }}^{2}$ = 0.27, β = 0.17; PCom: p = 0.155, ${{\upeta }}^{2}$ = 0.42, β = 0.26; PSRC: p = 0.449, ${{\upeta }}^{2}$ = 0.22, β = 0.11; PSRT: p = 0.559, ${{\upeta }}^{2}$ = -0.17, β = -0.08), nor for interaction with EG (PAut: p = 0.548, ${{\upeta }}^{2}$ = 0.25, β = 0.46; PCom: p = 0.419, ${{\upeta }}^{2}$ = 0.34, β = 0.59; PSRC: p = 0.285, ${{\upeta }}^{2}$ = -0.46, β = -0.71; PSRT: p = 0.560, ${{\upeta }}^{2}$ = 0.25, β = 0.47). Please see Table 2 for the results regarding the others motivation regulation types

## Discussion

### HRQoL Between the Groups

With the results presented, our analysis could not demonstrate any general differences in the level of HRQoL between EG and CG, which contradicts our initial hypothesis. It can be seen that the values in all groups are above the mean values of the validation of the used instrument and corresponding Europe-wide studies [80]. Simultaneously, the results are in the same range as those of a large German study in a population of children and adolescents of the same age also using the KIDSCREEN questionnaire [93]. The only significant result in our models was that the EG shows a significant drop in HRQoL over time (cf. Supplementary Fig. 1). Both the absence of group differences and the decrease in the EG contradict our preliminary assumptions, which were developed on the basis of several empirical and theoretical publications. They also contradict the few previous studies that have suggested that psychosocial well-being improves in EOtC [60]. A comparison of the results of these studies and our own is obvious due to the similar setting. Despite large overlaps in content, a different instrument was used in this studies which is why a comparison is not unrestrictedly valid. However, alignment with these studies suggests that within well-being and HRQoL, a focus on social and psychosocial aspects may be worthwhile in the future [60]. Furthermore, one possible explanation for the observed drop in HRQoL in the EG could be due to the seasons, as several empirical studies have already pointed out that winter time can have a negative impact on mental complaints and behavioural problems of younger people [94, 95]. As our T2 took place in (German) February, this effect may have been at play and the EG may have been even more influenced by this than the CG due to their increased exposure to nature. In contrast, Bølling et al. [60] were better able to account for that seasonal influence than ourself, as our study had to be shortened due to the COVID-19 pandemic. It furthermore seems conceivable that an additional positive influence on HRQoL via school-relevant aspects such as motivation and academic achievement could not be mapped in our forced limited time frame [46, 47].

### Motivational Regulation Between the Groups

With regard to motivational regulation, our statistical analysis revealed two key results that only partly confirm our hypotheses. First, the EG shows significant advantages over the CG in intrinsic and identified motivation - i.e. those forms of motivational regulation that can be referred to as self-determined (cf. Fig. 1 and Supplementary Fig. 2). The observed significant positive development of intrinsic motivational regulation over time thus seems to be mainly due to the EG, given the descriptive data. This is a very important finding because a steady decline in self-determined school motivation in students has already been documented in a large number of studies [96], even in studies comprising only one school year [64]. Here, the study results are consistent with previous research, which identified EOtC as a possible buffer against the drop of intrinsic motivation in regular classes [64, 97]. The results show even slightly higher intrinsic motivational regulation scores in the EG compared with Bølling et al. [64]. The moderate effect sizes are comparable between the two studies, despite different sample sizes and grouping strategies. We suspected in advance that possible changes in motivational regulation might be related to BPN. However, this could not be confirmed by our analysis. Possible explanations for the alternative source of the EG’s comparatively higher intrinsic and identified motivation are discussed in the following chapter.

The second central result of our analysis suggests that boys experience a negative development of external and introjected motivational regulation (cf. Supplementary Figs. 3 and 4). In principle, a decline in motivational values over time is no surprise considering previous research [45]. Additionally, differences between the genders in academic motivation (tending in favour of girls) have already been identified in the past. These differences in comparable aged populations on different academic motivation constructs in school may have been related to specific school subjects [98, 99] or focused on school routine as a whole [100]. Previous studies have also already pointed to gender differences in connection with these two types of controlled motivational regulation (e.g. [101]). In this case, the observed development, especially in external regulation, seems to be mainly due to the EG. In connection with the lack of a drop in intrinsic motivational regulation in the EG, one possible explanation could be that the boys’ motivational profile in this case shifts on the continuum in the direction of self-determined motivational regulation (cf. [102]). In principle, this mechanism of a displacement of external motivational regulation through a strong intrinsic motivational regulation could be valued as a positive finding, but it would have to be examined again in the future.

### Relationships Between Motivational Regulation, HRQoL and BPN

Looking at group differences in the associations of BPN and HRQoL or motivational regulation, our hypotheses could not be confirmed on a significant level. This applies to all models considered. On the verge of this, however, the results confirm the assumption of certain correlations over all groups. Following that, HRQoL is significantly positively associated with PSRC (cf. Fig. 2). However, such significant correlations could not be confirmed for the other BPN (cf. Supplementary Figs. 5-7). Although previous findings do not explicitly point to the enhanced importance of social relatedness for HRQoL over die other BPN, the fundamental connection between social factors and HRQoL has been researched and confirmed many times, especially in relation to the setting of school and peers of children and adolescents [30, 32]. Social interaction with classmates typically exceeds that with teachers, even in purely quantitative terms. This could be the reason why these correlations are only revealed for PSRC, but not for PSRT. From our point of view, it was conceivable that this effect could become even more apparent in the EOtC, but this was not confirmed by the analysis of our models. In contrast to this assumption, our MANOVA even indicates, that the CG shows significantly higher scores of PSRC.

Contrary to our preliminary assumption, our models could also neither confirm group differences in the correlations of the BPN with the different motivational regulation types nor confirm any correlations regarding this contructs over all groups. According to our MANOVA, the EG shows significantly higher levels of PAut and PCom and, as described above, higher levels of intrinsic motivational regulation. However, these two observations cannot be directly linked to each other on the basis of our models. Thus, our original hypothesis of an (positive) association of these constructs cannot be confirmed. However, we still see our results partly in the tradition of previous studies that suggest a positive relationship between autonomy and competence in particular and motivational regulation. Thus, when looking at intrinsic and identified motivation, these two BPN show a higher correlation coefficient than the facets of social relatedness. At least for the EG, also on a descriptive level, a tendential relationship cannot be completely discarded, even if not on a significant level (cf. Supplementary Figs. 8-11).

A possible, but at this stage speculative, alternative explanation for the increased levels of intrinsic and identified motivation in EG could be the teacher’s organisation of the lesson, which would not necessarily have to involve the promotion of PAut or PCom. Thus, it is known from school practice that particularly those curricular contents are used for EOtC that allow for designing a particularly vivid, exciting and experimental lesson. This could automatically increase students’ self-determined motivational regulation types of school motivation. Teachers who show a particularly high level of enthusiasm and enjoyment and who point out daily life relevance may opt for EOtC more frequently. These characteristics have been associated positively with school motivation of the respective students in previous studies [103–105] and could also exist independently of the EOtC and without the explicit support and satisfaction of PAut or PCom.

### Limitations and Future Directions

This study has some general methodological limitations. With our small sample size, the presented findings must be considered exploratory. Given this impression, we do not claim to generalise our findings. Rather, our study might provide information on how to better design future studies in the field of EOtC: Accordingly, the expansion to more enquiries and seasons would offer the chance to capture and reliably classify the effects of these via implementation as random effects on a statistical level [106]. Second, no random assignment of the participants to the groups could be realised in this study because we were dependent on the assignment by the parents. Future considerations should also pay attention to an even distribution in the sample, especially if gender effects are conceivable or expected as shown in our analyses regarding HRQoL. Furthermore, existing difficulties and challenges in capturing HRQoL - possibly but not necessarily related to social factors - in this population should be considered in future research [107–109]. In this respect, an even more elaborate selection of instruments and survey methods could possibly contribute to a breakthrough in this context and generate consistent results.

Overall, the basic relationships presented by the SDT between HRQoL, motivational regulation and BPN seem clear at the theoretical level and increasingly more empirical evidence supports them. This pilot study provided evidence for application in EOtC. Subsequent studies should test these relationships by enriching them with further variables (e.g. previous experience through forest kindergarten or socio-economic status) in elaborated statistical procedures, e.g. structural equation approaches. Especially the inclusion of academic achievements as a covariate seems unavoidable if the relationship between motivational regulation and HRQoL is to be examined in detail. Furthermore, when considering an influence of the BPN on HRQoL, a potentially negative effect due to an explicit non-satisfaction of the BPN should also be considered in the future.

## Conclusions

For the first time, we measured HRQoL in the EOtC and compared different groups over a longer period of time in a setting that is crucial for child development. As a curricular concept, EOtC could evolve its impact via long-term effects. The example of EOtC shows how the implementation of innovative concepts in schools could contribute to improving the rather poor situation in terms of mental health. Based on SDT multiple approaches exist, that could contribute to HRQoL of students by strengthening the satisfaction of BPN and motivational regulation, as shown by our study. Thus far, schools have only activated very limited capacities to support this [41]. To obtain well-founded results in mental health-related outcomes, such as HRQoL or BPN in the coming years, it is also necessary to constantly adapt the methodological approach in conducting studies to difficulties that arise. Only if it is possible to investigate relevant topics with suitable methods in significant settings in the future, the health of children and adolescents will be influenced positively on the long run.

## Supplementary Information

**Additional file 1.**

## Data Availability

The datasets used and/or analysed during the current study are available from the corresponding author on reasonable request.

## Abbreviations

WHO: World Health Organisation
PA: Physical activity
SDT: Self-determination theory
HRQoL: Health-related quality of life
BPN: Basic Psychological Needs
PCom: Perceived competence
PAut: Perceived autonomy
PSRC: Perceived social relatedness with classmates
PSRT: Perceived social relatedness with teachers
EOtC: Education Outside the Classroom
SB: Sedentary behaviour
CG: Control group
EG: Experimental group
BPNS: Basic Psychological Needs Scale
LMM: Linear mixed-effects model
