# Supplementary material for: Health-related quality of life, motivational regulation and Basic Psychological Need Satisfaction in Education Outside the Classroom: an explorative longitudinal pilot study
Source: BMC Public Health. 2022 Jan 8;22:49. doi: 10.1186/s12889-021-12450-9 (PMC8742160; doi:10.1186/s12889-021-12450-9)
Supplement: Supplementary file 1 — Additional file 1. [file 12889_2021_12450_MOESM1_ESM.docx]

**Supplementary Material:**

“Health-related Quality of Life, Motivational Regulation and Basic Psychological Need Satisfaction in Education Outside the Classroom: An Explorative Longitudinal Pilot Study”

**Characteristics of Education Outside the Classroom (EOtC) and Outdoor Teaching**

EOtC in the form it was conducted at our partner school is inspired by the formats that have been successfully conducted around the world for many years. According to the underlying understanding, all out-of-school places can be considered as places for teaching. Natural spaces in particular are frequently used in regularly implemented concepts. Here, mostly the existing natural materials on site are used to obtain seating, writing supports and teaching materials (1, 2). Those approaches are characterised by student-centred, but teacher-facilitated learning, as well as a lot of experimental, self-determined and real-life practical learning in most of the EOtC-receiving groups and classes (3, 4). This concept is nevertheless closely aligned to the curriculum and, in combination with normal indoor lessons, could contribute to improved learning (5, 3). It can be assumed that the removal of all spatial boundaries and old established structures in the classroom contributes to a restructuring of the learning process and interpersonal relationships with many at least theoretically reasonable effects on the children (3, 6).

**Supplementary Table 1** Model-fit parameters

| **Model** |  | **DF** | **AIC** | **p-value** |
| --- | --- | --- | --- | --- |
| 1 | Lme ( Intrinsic ~ (Paut + Pcom + PSRC + PSRT) * Gender * Enquiry * Group, random = ~ 1 \| Code)  Lme ( Intrinsic ~ (Paut + Pcom + PSRC + PSRT + Gender + Group) * Enquiry + (Paut + Pcom + PSRC + PSRT) * Group, random = ~ 1 \| Code) | 42  20 | 295.08  261.59 | 0.856 |
| 2 | Lme ( Identified ~ (Paut + Pcom + PSRC + PSRT) * Gender * Enquiry * Group, random = ~ 1 \| Code)  Lme ( Identified ~ (Paut + Pcom + PSRC + PSRT + Gender + Group) * Enquiry + (Paut + Pcom + PSRC + PSRT) * Group, random = ~ 1 \| Code) | 42  20 | 285.95  253.23 | 0.826 |
| 3 | Lme ( Introjected ~ (Paut + Pcom + PSRC + PSRT) * Gender * Enquiry * Group, random = ~ 1 \| Code)  Lme ( Introjected ~ (Paut + Pcom + PSRC + PSRT + Gender + Group) * Enquiry + (Paut + Pcom + PSRC + PSRT) * Group, random = ~ 1 \| Code) | 42  20 | 301.75  290.95 | 0.029 |
| 4 | Lme ( External ~ (Paut + Pcom + PSRC + PSRT) * Gender * Enquiry * Group, random = ~ 1 \| Code)  Lme ( External ~ (Paut + Pcom + PSRC + PSRT + Gender + Group) * Enquiry + (Paut + Pcom + PSRC + PSRT) * Group, random = ~ 1 \| Code) | 42  20 | 277.33  260.61 | 0.105 |
| 5 | Lme ( OverallHRQoL ~ (Paut + Pcom + PSRC + PSRT) * Gender * Enquiry * Group, random = ~ 1 \| Code)  Lme ( OverallHRQoL ~ (Paut + Pcom + PSRC + PSRT + Gender + Group) * Enquiry + (Paut + Pcom + PSRC + PSRT) * Group, random = ~ 1 \| Code) | 42  20 | 828.42  709.07 | <0.001 |

DF = Degrees of Freedom; AIC = Akaike Information Criterion; Intrinsic = Intrinsic Motivational Regulation; Identified = Identified Motivational Regulation; Introjected = Introjected Motivational Regulation; External = External Motivational Regulation; PAut = Perceived Autonomy; PCom = Perceived Competence; ; PSRC = Perceived Social Relatedness with Classmates; PSRT = Perceived Social Relatedness with Teachers; OverallHRQoL = Overall Health-Related Quality of Life

**Supplementary Table 2** Results of multivariate analysis of variance regarding group differences in BPN scores, p-values (p), effect sizes ($\eta^{2}$), F-statistics (F) and confidence intervals (CI)

|  | **p** | $\boldsymbol{\eta}^{\mathbf{2}}$ | **F** | **CI*** |
| --- | --- | --- | --- | --- |
| **PAut** | **<0.05** | 0.17 | 6.276 | -0.19 / 0.53 |
| **PCom** | **<0.01** | 0.43 | 11.218 | -0.10 / 0.61 |
| **PSRT** | 0.778 | 0.15 | 3.160 | -0.18 / 0.40 |
| **PSRC** | **<0.001** | 0.50 | 14.547 | -0.37 / 0.20 |

*95%, Lower / Upper confidence interval; PAut = Perceived Autonomy; PCom = Perceived Competence; PSRC = Perceived Social Relatedness with Classmates; PSRT = Perceived Social Relatedness with Teachers

**Supplementary** **Table 3** Statistical characteristics for the satisfaction of the BPN of PSRT, PSRC, PAut and PCom between groups and enquiries.

|  | | | **T 1** | | | |  | **T 2** | | | | |
| --- | --- | --- | --- | --- | --- | --- | --- | --- | --- | --- | --- | --- |
|  |  |  | **PSRT** | **PSRC** | **PAut** | **PCom** |  | | **PSRT** | **PSRC** | **PAut** | **PCom** |
| **EG** | **Girls** | **M** | 4.18 | 4.25 | 3.86 | 4.15 |  | | 4.27 | 4.14 | 4.09 | 4.19 |
|  |  | **SD** | 0.54 | 0.76 | 0.68 | 0.68 |  | | 0.39 | 0.62 | 0.38 | 0.32 |
|  |  | **CI*** | 3.86 /  4.5 | 3.8 /  4.7 | 3.46 /  4.26 | 3.75 /  4.55 |  | | 4.04 /  4.5 | 3.78 /  4.5 | 3.87 /  4.28 | 4.0 /  4.38 |
|  | **Boys** | **M** | 4.37 | 4.38 | 4.10 | 4.21 |  | | 4.29 | 4.31 | 4.18 | 4.34 |
|  |  | **SD** | 0.60 | 0.39 | 0.61 | 0.52 |  | | 0.76 | 0.53 | 0.66 | 0.51 |
|  |  | **CI*** | 4.04 /  4.96 | 4.18 /  4.58 | 4.42 /  3.78 | 4.48 /  3.94 |  | | 3.89 /  4.69 | 4.03 /  4.59 | 3.84 /  4,58 | 4.08 /  4.6 |
|  | **All** | **M** | 4.31 | 4.34 | 3.99 | 4.18 |  | | 4.28 | 4.23 | 4.14 | 4.27 |
|  |  | **SD** | 0.58 | 0.60 | 0.65 | 0.60 |  | | 0.61 | 0.58 | 0.54 | 0.43 |
|  |  | **CI*** | 4.08 /  4,54 | 4.11 /  4.57 | 3.74 /  4.24 | 3.95 /  4.41 |  | | 4.04 /  4.52 | 4.0 /  4.46 | 3.93 /  4.35 | 4.1 /  4.44 |
| **CG** | **Girls** | **M** | 4.13 | 4.42 | 3.95 | 4.03 |  | | 4.13 | 4.52 | 3.68 | 3.81 |
|  |  | **SD** | 0.77 | 0.50 | 0.67 | 0.50 |  | | 0.78 | 0.70 | 0.67 | 0.67 |
|  |  | **CI*** | 4.52 /  3.74 | 4.27 /  4.67 | 3.61 /  4.29 | 3.78 /  4.28 |  | | 3.74 /  4.52 | 4.14 /  4.88 | 3.34 /  4.02 | 3.47 /  4.15 |
|  | **Boys** | **M** | 3.81 | 4.64 | 3.89 | 3.91 |  | | 3.44 | 4.35 | 3.57 | 3.77 |
|  |  | **SD** | 0.63 | 0.44 | 0.47 | 0.63 |  | | 0.74 | 0.68 | 0.61 | 0.59 |
|  |  | **CI*** | 3.57 /  4.05 | 4.47 /  4.81 | 3.71 /  4.07 | 3.67 /  4.15 |  | | 3.15 /  3.73 | 4.09 /  4.61 | 3.34 /  3.8 | 3.54 /  4.0 |
|  | **All** | **M** | 3.93 | 4.56 | 3.91 | 3.96 |  | | 3.59 | 4.41 | 3.52 | 3.79 |
|  |  | **SD** | 0.70 | 0.48 | 0.56 | 0.59 |  | | 0.82 | 0.69 | 0.63 | 0.62 |
|  |  | **CI*** | 3.72 /  4.14 | 4.41 /  4.71 | 3.74 /  4.08 | 3.16 /  4.14 |  | | 3.34 /  3.84 | 4.2 /  4.62 | 3.33 /  3.71 | 3.6 /  3.98 |
| **All** | | **M** | 4.07 | 4.48 | 3.94 | 4.04 |  | | 3.85 | 4.34 | 3.75 | 3.97 |
|  |  | **SD** | 0.68 | 0.54 | 0.60 | 0.60 |  | | 0.81 | 0.66 | 0.65 | 0.61 |
|  |  | **CI*** | 3.91 /  4.23 | 4.35 /  4.61 | 3.8 /  4.08 | 3.9 /  4.18 |  | | 3.66 /  4.04 | 3.74 /  4.5 | 3.59 /  3.91 | 3.82 /  4.12 |

PSRT = Perceived social relatedness with teachers; PSRC = Perceived social relatedness with classmates; PCom = Perceived Competence; PAut = Perceived Autonomy; M = mean; SD = standard deviation; CI = confidence interval; * 95%, Lower / Upper CI

**
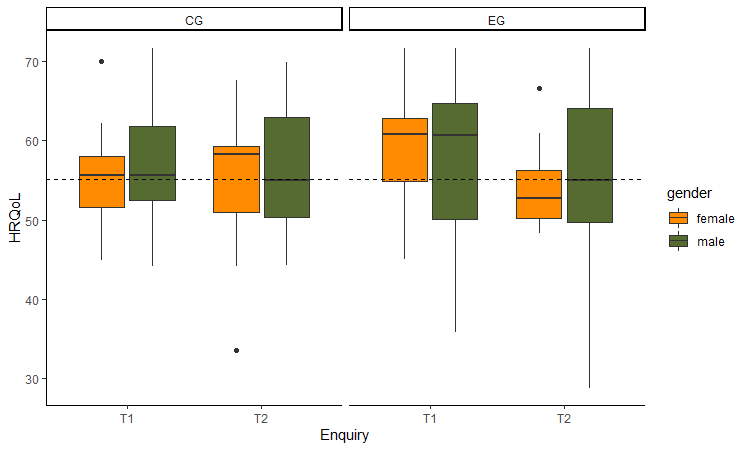
**

**Supplementary Fig. 1** HRQoL scores between groups, genders and time points with reference line representing a large-scale representative study on German children and adolescents (6)

HRQoL = Health-Related Quality of Life; CG = Control Group; EG = Experimental Group; T1 = First time point; T2 = Second time point

**
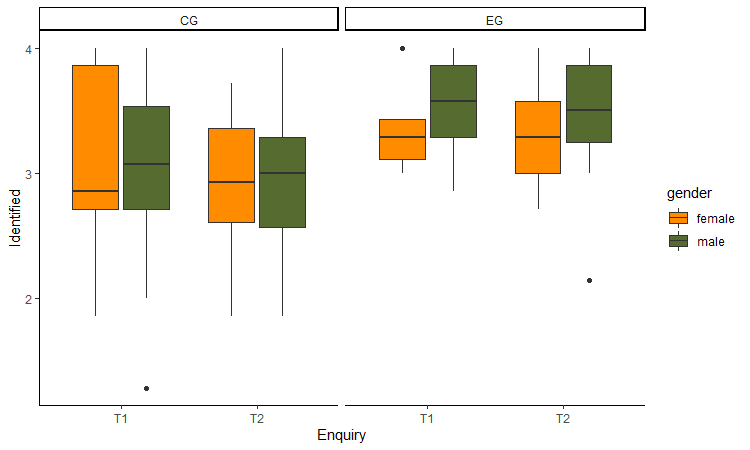
**

**Supplementary Fig. 2** Scores of identified motivational regulation between groups, genders and time points

Identified = Identified Motivational Regulation; CG = Control Group; EG = Experimental Group; T1 = First time point; T2 = Second time point

**
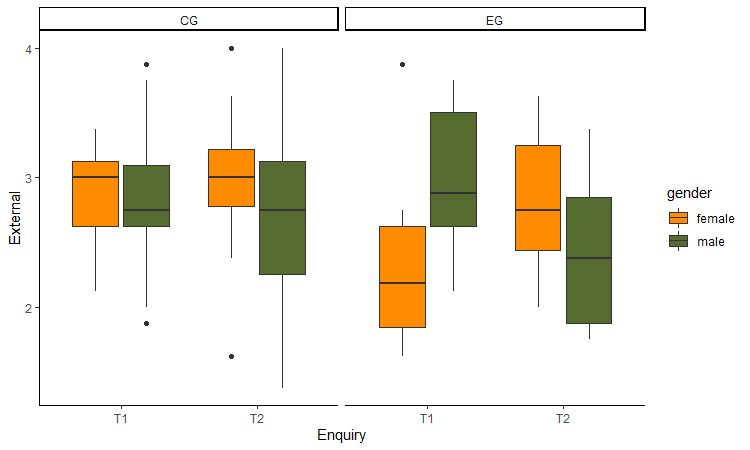
**

**Supplementary Fig. 3** Scores of external motivational regulation between groups, genders and time points

External = External Motivational Regulation; CG = Control Group; EG = Experimental Group; T1 = First time point; T2 = Second time point

**
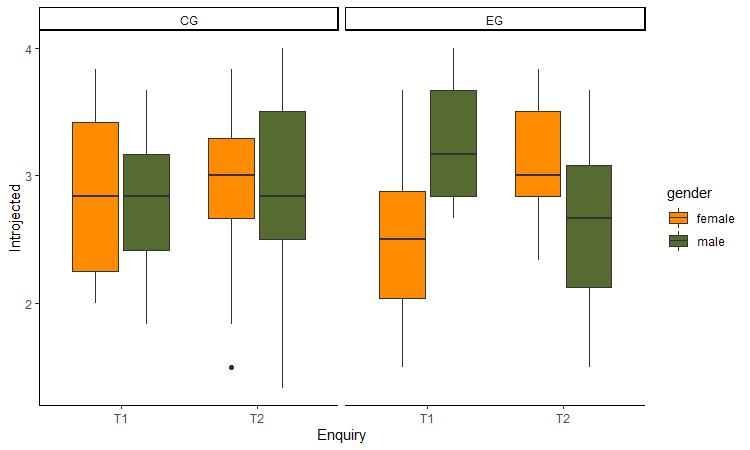
**

**Supplementary Fig. 4** Scores of introjected motivational regulation between groups, genders and time points

Introjected = Introjected Motivational Regulation; CG = Control Group; EG = Experimental Group; T1 = First time point; T2 = Second time point

**
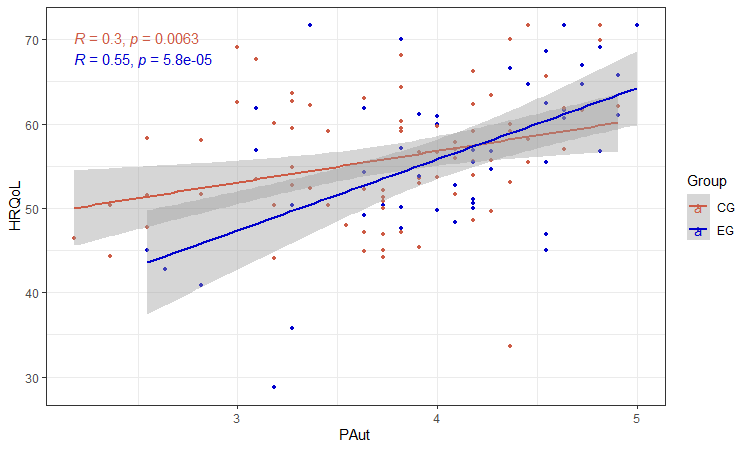
**

**Supplementary Fig. 5** PAut and HRQoL between groups over all time points

HRQoL = Health-related quality of life; PAut = Perceived autonomy; CG = Control group; EG = Experimental group

**
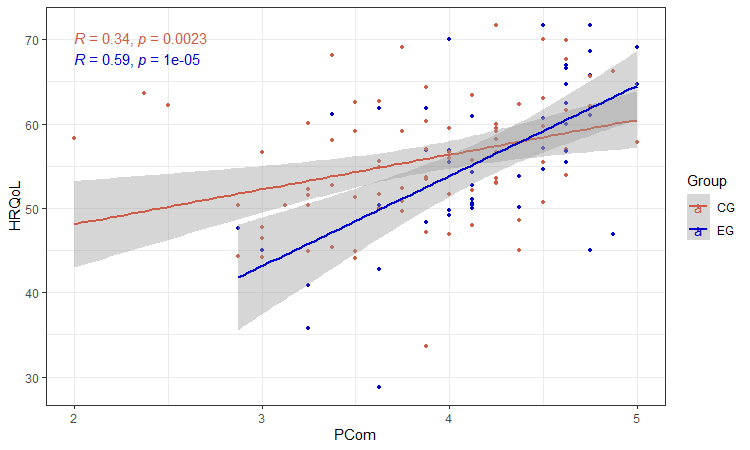
**

**Supplementary Fig. 6** PCom and HRQoL between groups over all time points

HRQoL = Health-related quality of life; PCom = Perceived competence; CG = Control group; EG = Experimental group

**
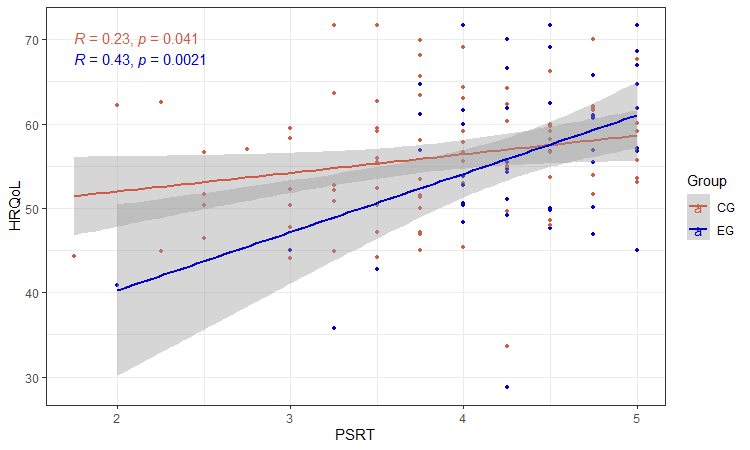
**

**Supplementary Fig. 7** PSRT and HRQoL between groups over all time points

HRQoL = Health-related quality of life; PSRT = Perceived social relatedness with teachers; CG = Control group; EG = Experimental group

**
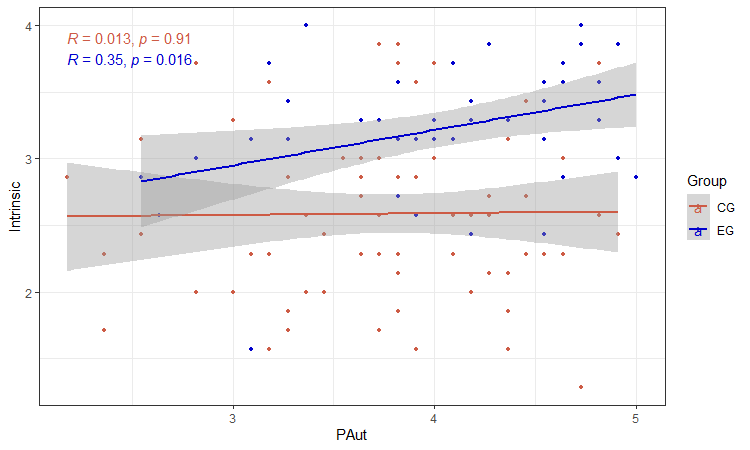
**

**Supplementary Fig. 8** PAut and intrinsic motivational regulation between groups over all time points

Intrinsic = Intrinsic motivational regulation; PAut = Perceived autonomy; CG = Control group; EG = Experimental group

**
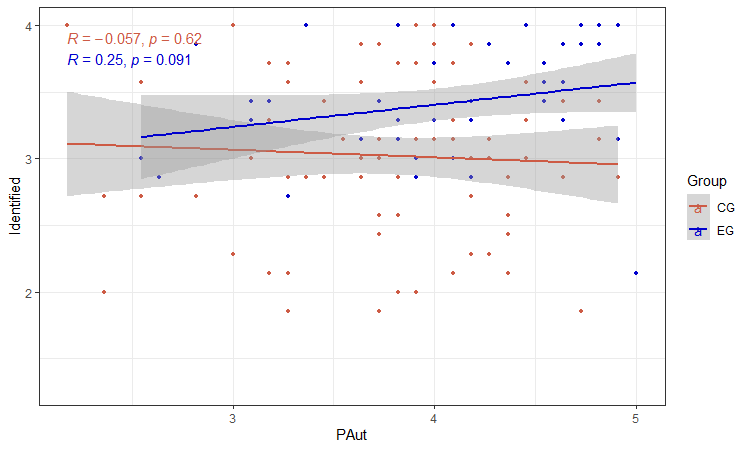
**

**Supplementary Fig. 9** PAut and identified motivational regulation between groups over all time points

Identified = Identified motivational regulation; PAut = Perceived autonomy; CG = Control group; EG = Experimental group

**
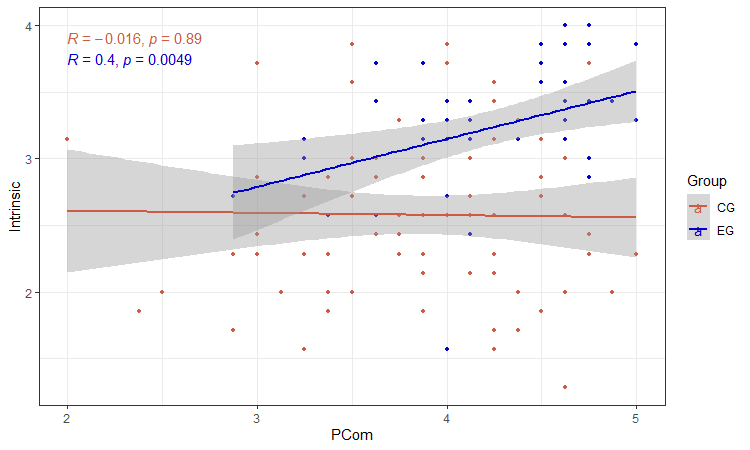
**

**Supplementary Fig. 10** PCom and intrinsic motivational regulation between groups over all time points

Intrinsic = Intrinsic motivational regulation; PCom = Perceived competence; CG = Control group; EG = Experimental group

**
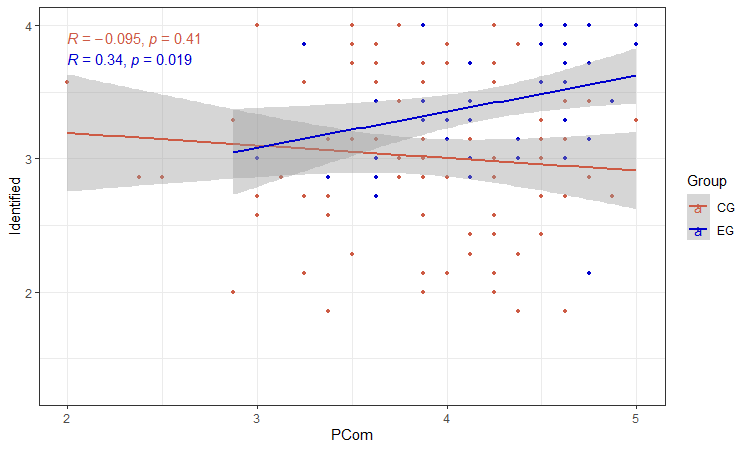
**

**Supplementary Fig. 11** PCom and identified motivational regulation between groups over all time points

Identified = Intrinsic motivational regulation; PCom = Perceived competence; CG = Control group; EG = Experimental group

**References**

1. Beames S, Higgins P, Nicol R. Learning outside the classroom: Theory and guidelines for practice. London: Routledge; 2012.
2. Bentsen P, Jensen F, Mygind E, Randrup T. The extent and dissemination of udeskole in Danish schools. Urban For Urban Green. 2010;9(3):235–243. <https://doi.org/10.1016/j.ufug.2010.02.001>.
3. von Au J. Outdoor Education an Schulen in Dänemark, Schottland und Deutschland – kompetenzorientierte und kontextspezifische Einflüsse auf Intentionen und Handlungen von Erfahrenen Outdoor Education-Lehrpersonen [Outdoor Education in Danish, Scottisch and German Schools - Competence Oriented and Context Specific Influences on Intentions and Actions of Experienced Outdoor Education Teachers]. Heidelberg; 2016.
4. Bentsen P, Mygind L, Elsborg B, Nielsen G, Mygind E. Education outside the classroom as upstream school health promotion: ‘adding-in’ physical activity into children’s everyday life and settings. Scand J Public Health. 2021;1(9). <https://doi.org/10.1177/1403494821993715>.
5. Barfod K, Eibye-Ernst N, Mygind L, Bentsen P. Increased provision of udeskole in Danish schools: an updated national population survey. Urban For Urban Green. 2016;20:277-281. <https://doi.org/10.1016/j.ufug.2016.09.012>.
6. von Au J. Outdoor Days - Draußentage - Lernen mit Herz, Hand und viel Verstand [Learning with passion, hand and a clear mind]. Pädagogik. 2018;4(18):10–13.
7. Ellert U, Brettschneider A-K, Ravens-Sieberer U, Group KS. Gesundheitsbezogene Lebensqualität bei Kindern und Jugendlichen in Deutschland [Health-related quality of life among children and adolescents in Germany]. Bundesgesundheitsbl. 2014;57(7):798-806. <https://doi.org/10.1007/s00103-007-0244-4>.
